# Inadequate Knowledge, Attitude and Practices about Second-Hand Smoke among Non-Smoking Pregnant Women in Urban Vietnam: The Need for Health Literacy Reinforcement

**DOI:** 10.3390/ijerph17103744

**Published:** 2020-05-25

**Authors:** Giap Van Vu, Chau Quy Ngo, Phuong Thu Phan, Lan Phuong Thi Doan, Toan Thi Nguyen, Mai Hong Nguyen, Diep Ngoc Nguyen, Nguyen Thao Thi Nguyen, Huong Lan Thi Nguyen, Chi Linh Hoang, Linh Gia Vu, Carl A. Latkin, Bach Xuan Tran, Roger C. M. Ho, Cyrus S. H. Ho

**Affiliations:** 1 Department of Internal Medicine, Hanoi Medical University, Hanoi 100000, Vietnam; vuvangiap@hmu.edu.vn (G.V.V.); ngoquychaubmh@gmail.com (C.Q.N.); thuphuongdr@gmail.com (P.T.P.); nguyenthitoanmika.hmu@gmail.com (T.T.N.); nguyenhongmai1996@gmail.com (M.H.N.); 2 Respiratory Center, Bach Mai Hospital, Hanoi 100000, Vietnam; phuonglandoanthibm@yahoo.com.vn; 3 Institute for Global Health Innovations, Duy Tan University, Da Nang 550000, Vietnam; nguyenngocdiep7@duytan.edu.vn; 4 Faculty of Pharmacy, Duy Tan University, Da Nang 550000, Vietnam; 5 Duke University School of Medicine, Duke University, Durham, NC 27710, USA; thao.nguyen@duke.edu; 6 Faculty of Nursing, Duy Tan University, Da Nang 550000, Vietnam; 7 Center of Excellence in Behavioral Medicine, Nguyen Tat Thanh University, Ho Chi Minh City 700000, Vietnam; chi.coentt@gmail.com; 8 Center of Excellence in Evidence-Based Medicine, Nguyen Tat Thanh University, Ho Chi Minh City 700000, Vietnam; linh.coentt@gmail.com; 9 Bloomberg School of Public Health, Johns Hopkins University, Baltimore, MD 21205, USA; bach.ipmph@gmail.com (B.X.T.); carl.latkin@jhu.edu (C.A.L.); 10 Institute for Preventive Medicine and Public Health, Hanoi Medical University, Hanoi 100000, Vietnam; 11 Department of Psychological Medicine, Yong Loo Lin School of Medicine, National University of Singapore, Singapore 119228, Singapore; pcmrhcm@nus.edu.sg; 12 Institute for Health Innovation and Technology (iHealthtech), National University of Singapore, Singapore 119077, Singapore; 13 Department of Psychological Medicine, National University Hospital, Singapore 119074, Singapore; cyrushosh@gmail.com

**Keywords:** Vietnam, second-hand smoke (SHS), pregnant women, knowledge, attitude, practice, KAP, smoke-free legislation

## Abstract

The rate of exposure to second-hand smoke (SHS) is relatively high in several countries, including Vietnam, and health issues related to SHS have worsened in recent years, especially for pregnant women and their infants. Enhancement of knowledge, attitude, and practice (KAP) scores of pregnant women in Vietnam could raise practical interventions to protect their health and reduce complications of SHS. A cross-sectional study of 432 pregnant women who came to the Obstetrics Department of Bach Mai Hospital, Hanoi, Vietnam for antenatal care was conducted in 2016 to collect information about their KAP related to SHS. Composite mean scores from survey questions assessing their KAP were calculated on a 10-point scale, finding mean scores of 4.19, 7.45, and 4.30, respectively. Higher scores indicated better knowledge, attitude, and practice. Generalized linear models identified that age, occupation, living place, and sources of information were associated with SHS-related KAP. Findings from this study indicate that suitable programs related to SHS should be implemented to improve and reinforce health literacy to both mothers and smokers to reduce the harmfulness of smoking on women and their infants’ health.

## 1. Introduction

Second-hand smoke (SHS) is well-recognized as a public health concern, especially for vulnerable populations such as pregnant women. According to a WHO report in 2009, about 35% of pregnant women were affected by SHS globally [1]. The global population has sustained SHS exposure at high rates as well; according to a study collecting data from 192 countries, estimated rates of children, male non-smokers, and female non-smokers who were exposed to SHS were 40%, 33%, and 35%, respectively [2]. The rate of sustained SHS exposure has decreased in some countries including the USA [3], Japan [4], Korea [5], and Scotland [6], due to increased smoking regulations. However, in other countries, this rate has remained high, for example at 57.1% in Germany [7] and nearly 50% in Bangladesh [8]. Among pregnant women, this prevalence appears to be increasing over time, as demonstrated in a meta-analysis in 2014 which illustrated an even higher prevalence of pregnant women exposed to tobacco smoke (from more than 50% to 85%, depending on country) [9]. SHS exposure during pregnancy causes severe health problems for mothers, including depressive symptoms, cardiovascular diseases, hypertension together with concerned respiratory issues (e.g., asthma, chronic bronchitis, emphysema) [2,10,11,12,13], pregnancy complications including spontaneous abortion, placental abruption, and low birth weight [14,15,16,17], and even longer-term consequences on the infant, including heightened risk of sudden infant death syndrome [18]. Given the high rates of SHS exposure globally, understanding current perceptions of SHS is crucial in order to design appropriate interventions to address this public health issue.

Globally, improving knowledge, attitude, and practice (KAP) is considered an effective solution to reducing exposure to SHS [19,20]. Prior studies in other middle-income countries, such as China [21,22] and Argentina, [23] have indicated significantly low KAP among pregnant women. In particular, low KAP scores were observed among individuals with younger age, low education, and some socioeconomic factors [24,25,26], though these factors varied across study settings, indicating the need for region-specific evidence to develop contextualized interventions [9].

In Vietnam, there have been increasing efforts to regulate smoking in recent years, including a 2005 law that prohibited smoking in certain public places. Per the 2015 Global Adult Tobacco Survey (GATS), the rate of adult tobacco use in Vietnam is 22.5% among the general population, a high percentage despite decreases in recent years [27]. A study in 2010 found that general knowledge of the risks of SHS among the Vietnamese population was 83%, though knowledge of specific diseases related to tobacco smoking was lower at 51.5% [28]. Yet, despite this progress, the rates of SHS exposure among Vietnamese pregnant women have not demonstrated strong improvement. Previous studies have shown that rates of SHS exposure among pregnant Vietnamese women have remained high, with similar patterns occurring in other low- and middle-income countries [29,30,31]. According to a recent study in 2019, the rate of pregnant Vietnamese women who endured SHS exposure in their lifetime was 92.6%, and during the last 30 days of pregnancy, the rate was 64.5% [32], relatively high. However, data on KAP regarding SHS in this population are lacking. This study explores the KAP of pregnant women who are in vulnerable populations and examines the factors that influence their KAP towards SHS exposure during pregnancy.

Even though existing studies have focused on the health burden from SHS globally [2,33,34] and among the Vietnamese population [28,35,36], there are not many studies on KAP of women about SHS exposure during their pregnancy, which is a sensitive period. Therefore, this study makes a major contribution to research on KAP regarding SHS among high-risk pregnant women, and explores applicable policy and implementation on smoking law.

## 2. Materials and Methods

### 2.1. Study Designs

During 2 months in 2016 (July and August), a cross-sectional study was conducted among 432 pregnant women who came to the Obstetrics Department of Bach Mai Hospital, Hanoi—one of the largest hospitals in Vietnam. The Obstetrics Department has about 6700 pregnant women who come for antenatal care per year. In this study, we used convenience sampling to recruit pregnant women. Inclusion criteria included: (1) they were at least 18 years old; (2) they were able to answer the interview; and (3) they declared their agreement through written informed consent. This study was a sub-analysis of data collected as part of a study examining the prevalence and sources of SHS exposure among pregnant women in Vietnam [32].

### 2.2. Measurements

Thirteen medical students and nurses of the Respiratory Center of Bach Mai Hospital collected the data from the pregnant women, after receiving a 20 h training workshop. Data regarding pregnancy status was collected through the regular booking system for all women requesting an appointment to the Obstetrics Department. Data collectors approached pregnant women, who were identified by the booking system as pregnant, and reviewed their eligibility and consent. In order to assure their confidentiality and comfort, they were invited into a private room. All participants were introduced to the purposes of the study and advised about their rights to withdraw at any time without any influence on their current care. Data collectors interviewed participants to compile information about their demographics (e.g., age, educational level, occupation, and living place) and pregnancy (e.g., gestation week), as well as sources of information about SHS through a questionnaire. Through literature review and expert consultations, we synthesize these following questions to measure knowledge, attitude, and practices of participants. The full survey is available in Table A1 of Appendix A.

#### 2.2.1. Knowledge

Pregnant women were asked to answer the following questions to evaluate their knowledge: What is the definition of SHS? What are the health effects of SHS on pregnant women? What are the health effects of SHS on infants/fetuses? What kinds of facilities completely prohibit smoking indoors and outdoors according to the law? What kinds of facilities completely prohibit smoking indoors according to the law? Participants were given one point for each question they were able to answer correctly; for answers with multiple responses, they were given one point if they were able to select all correct responses. The total knowledge score ranged from 0 to 5; a higher score demonstrated a higher level of knowledge. Cronbach’s alpha scores were 0.843 and 0.72 for the questions about health effect and compliance, respectively.

#### 2.2.2. Attitude

Participants were asked to express their opinions about smoking at home/workplace/public areas (should be/not be banned). If they answered that smoking should not be allowed at home/workplace/public areas, they earned 1 point per place. The total attitude score was from 0 to 3, in which a higher score indicated a more negative attitude toward SHS. Cronbach’s alpha score was 0.51.

#### 2.2.3. Practices

Regarding practices, we asked pregnant women to report whether they reminded smokers about the smoke-free law at home/workplace/public areas. Each question was scored from 0 to 4, representing never to always. We also asked them to report whether they had taken action to prevent smoking behavior at these places. If they did, they gained one point for each question. The total practice score was from 0 to 15 with higher scores demonstrating higher levels of practice. Cronbach’s alpha score was 0.75.

### 2.3. Statistical Analysis

Stata software (STATA 14.0, College Station, TX, USA) was applied to analyze collected data. Statistical significance was detected if a p-value was less than 0.05. The total knowledge, attitude, and practice scores were transformed to a 10-point scale for better interpretation. Generalized linear models using the Gaussian family and identity link were employed to identify factors associated with knowledge, attitude, and practice scores. The potential associated factors included socio-demographic characteristics and sources of information. The forward stepwise selection was applied to formulate the reduced model that only contained independent variables having a log-likelihood ratio test p-value less than 0.2.

### 2.4. Ethical Approval

The Vietnam Respiratory Society Scientific and Ethics Committee has previewed and approved the study protocol (10-QD/VNRS).

## 3. Results

Demographic characteristics of participated women are summarized in Table 1. In brief, among 432 pregnant women, 46.3% of them were aged 26–30 years old. The majority of women had above high school educational level (79.6%) and were currently employed (60.7%). Most participants were living in urban areas (86.1%). The rate of pregnant women at 30–37 gestation weeks and <30 gestation weeks were 46.3% and 37.7%, respectively [32]. In addition, data on the main sources of information about SHS for pregnant women indicated that television was the primary source, following by news/magazines, and the internet; leaflets were the least common ones.

Knowledge about SHS is shown in Table 2. Most of the respondents correctly defined SHS (62.0%) and identified facilities where smoking is completely prohibited indoors and outdoors according to the law. The majority of women answered correctly that SHS could cause pulmonary diseases (92.1%), lung cancer (80.8%), and miscarriage/complications (74.8%). Moreover, they correctly answered that exposure to SHS could cause miscarriage/stillbirth (76.4%) and preterm birth/low birth weight (76.9%). The mean knowledge score was 4.19/10 (SD = 2.12).

Regarding attitude toward SHS, the mean attitude score was 7.45/10 (SD = 3.05). The majority of them recommended that smoking should be banned at home (85.7%), workplace (70.4%), and in public areas (67.6%) (Table 3).

As shown in Table 4, women more frequently reminded smokers at home to stop smoking, compared to other places such as workplaces and public areas. Likewise, the proportion of individuals who took action to stop smoking at home was 99.3%, which was much higher than that in the workplace (49.3%) and public areas (21.1%). The mean score for practices was 4.30/10 (SD = 2.39).

Table 5 depicts the factors associated with knowledge, attitude, and practice scores regarding SHS among pregnant women. People aged 31–35 (coef. = 0.51; 95% CI = 0.02–0.99), employed (coef. = 0.66; 95% CI = 0.22–1.11), and who receive information from the news/magazines about SHS (coef. = 0.65; 95% CI = 0.22–1.08) had significantly higher knowledge scores than those aged 18–25, self-employed, and who do not receive information regarding SHS from the news/magazines, respectively. Meanwhile, increasing gestation week was correlated with lower knowledge score (coef. = −0.02; 95% CI = −0.03; −0.00).

Regarding attitude, living in rural areas (coef. = 0.99; 95% CI = 0.24–1.74) and using the internet (coef. = 0.68; 95% CI = 0.01–1.36) and loudspeaker (coef. = 1.17; 95% CI = 0.05–2.28) as sources of information significantly increased the attitude score. Meanwhile, being unemployed/housewife (coef. = −1.08; 95% CI = −1.76–−0.39), living in rural area (coef. = −0.60; 95% CI = −1.16–−0.04) and hearing about SHS from advertisements (coef. = −1.26; 95% CI = −2.39–−0.13) was associated with a lower practice score. Higher knowledge score was correlated with higher practice score (coef. = 0.13; 95% CI = 0.03–0.24).

## 4. Discussion

This study provides greater insight on KAP regarding SHS among high-risk pregnant women. Our findings reveal the low degree of knowledge (mean score 4.19/10, SD = 2.12) and practice (mean score 4.30/10, SD = 2.39) with a moderately high attitude (7.45/10, SD = 3.05) toward SHS among this population. Moreover, results of multivariate models suggested that age, occupation, living place, and sources of information were associated with SHS-related KAP, highlighting populations that should be given special attention in further interventions.

Despite good knowledge about general SHS-related consequences (e.g., pulmonary lung diseases, preterm birth, or miscarriage) among our sample, we still found major gaps in knowledge. Only a small percentage of pregnant women were aware of SHS’s harms regarding peptic ulcers, sexual dysfunction, risk of cardiovascular diseases, or various cancers. Similarly, the proportion of women with knowledge of the long-term negative effects of perinatal SHS exposure on infants was not high, especially regarding sudden infant death syndrome and infant mortality. Our results were comparable to previous studies in other developing countries [37,38] but lower than those in developed nations [39]. This knowledge deficiency highlights the need for further interventions to increase awareness of these lesser-known complications among this population [37,40,41,42].

Likewise, practices to prevent SHS by pregnant women were minimally observed, even though the majority felt annoyed when others smoked. Per our results, practices against SHS at home were the highest, followed by workplace with lowest rates in public areas. One possible explanation is that pregnant women might believe that the nearer the smoker is, the greater the health hazard during pregnancy. However, the distance between smokers and pregnant women was not the only important factor affecting pregnant women’s health [43]. Another explanation could be that women may feel more comfortable taking action in their own home and other familiar places, as opposed to public areas where they may not know the smoker. This result aligns with a previous study in Vietnam that found that only 69.8% of the population supported the ban on smoking in public places [44]. The law No. 09/2012/QH13 of Vietnam mandated the regulations of “no smoking in public places” and “no smoking at workplaces” and intended regulation of “increasing the tobacco tax” [45]. Furthermore, it has been shown that prohibiting smoking in public places has been largely ineffective in changing behaviors of frequent smokers because of its intangibility, or even, perhaps, lack of awareness of the law [46,47]. Though there have been designated smoke-free areas in Vietnam since 2005, data up to 2010 shows that smoking in public places still occurred quite commonly; (22.3%, 23.6%, 34.4%, 38.7%, 54.3%, 84.9%, and 92.6% were the rates of schools, healthcare facilities, public transport, government buildings, universities, restaurants, and bars/cafes/tea shops, respectively), and 71.3% of adults were victims of SHS [48]. As such, this data suggests the need for stricter enforcement of smoke-free areas—both by local authorities and by the general public. Our data suggests that familiarity increases the likelihood of pregnant women taking action to maintain smoke-free spaces—with women most uncomfortable taking action in public areas among strangers. This hesitancy could be mitigated through increased enforcement of smoke-free areas, as well as empowerment of the general public to take action alongside pregnant women to prevent smoking in those areas. Indeed, while pregnant women have the right to smoke-free spaces, it is not their sole responsibility to enforce regulations provided by the law.

Our data also demonstrates that practices against SHS are low among rural populations and the unemployed/housewives. This finding aligns with previous studies which have shown that rural populations support smoking as a traditional, popular behavior, as well as a leisure activity [49,50]. Moreover, low educational level and lack of information on health-related hazards due to smoking were also associated with less practical activities to personally quit smoking or prevent others from smoking [51,52,53,54]. These results also suggest that women at an earlier gestation period had less knowledge about the harms from SHS, highlighting the need to educate pregnant women earlier during their pregnancy. Recommendations from doctors in healthcare facilities and other information channels about the harmfulness of SHS to pregnant women and infants could be helpful to reduce exposure to SHS [55]. Other preconceptual and prenatal programs/workshops for women of reproductive age should be implemented more frequently to further educate regarding healthy behaviors [56]. Per our data, effective channels to disseminate information on SHS could be internet and community. Both of these channels have a wide range of influence in the community and as such, can be effective tools to spread health literacy on SHS [57,58,59].

As demonstrated in this study, interventions focusing on specific vulnerable populations such as pregnant women are needed. Firstly, we should make use of effective channels, such as internet and loudspeakers, to enhance the knowledge of pregnant women about the harmfulness of SHS. Secondly, educational programs about smoking and its hazards could be implemented to provide awareness and necessary actions to stop others smoking. These programs might be designed for specific groups. For example, education and smoking prevention programs have been implemented at school level and proven effective through the decrease of smoking prevalence among pupils and school staff [60,61]. Similarly, family-based programs could be effective for couples who plan to have babies or already have small children. Besides these, online education that supports smokers to discontinue smoking, and a smartphone mobile application, might help control/lessen the frequency of smokers, which could be helpful within the development of technology [62,63]. Thirdly, smoking regulations in Vietnam have not been sufficiently effective to reduce smoking. There should be a greater emphasis on raising awareness and enforcement to ensure that the regulations produce the intended results. Public health interventions should empower not only pregnant women, but also the general population, to take shared responsibility in enforcing smoke-free spaces and protect vulnerable populations.

This study has several limitations. Firstly, due to this study’s cross-sectional nature, we are unable to determine any causal relationships between independent and dependent variables. Secondly, the results may also not be generalisable to all pregnant women, because this is a sample of pregnant women attending a single hospital in Vietnam. Thirdly, this study focused only on KAP of pregnant women regarding SHS. Without a control group, such as non-pregnant women of reproductive age, we are limited in our ability to understand specifically the role of pregnancy in KAP regarding smoking among the pregnant women in our study. Although Cronbach’s alpha scores for knowledge and practices were high, the questionnaire has not been validated about its reliability yet, because the Cronbach’s alpha score for the attitudes was low, suggesting that the questions on attitudes are not reliable enough. Finally, a cross-sectional study might lead to risk of recall-bias, where they could not remember exactly their attitude and/or practices over two months. Nonetheless, well-trained interviewers helped us minimize the bias.

## 5. Conclusions

In conclusion, our data indicated inadequate KAP of pregnant women in Vietnam about SHS and its health-related hazards. Suitable programs related to SHS should be implemented to improve and reinforce health literacy and practical reactions to smokers, to reduce the harmfulness of smoking to women and their infants’ health. The policymakers should enforce the smoke-free law to protect pregnant women and the general population from SHS.

## Figures and Tables

**Table 1:** Demographic characteristics of pregnant women.

Characteristics	Total	
	n	%
**Total**	432	100.0
**Age group**		
18–25	118	27.3
26–30	200	46.3
31–35	79	18.3
>35	35	8.1
**Education attainment**		
<High school	16	3.7
High school	72	16.7
>High school	344	79.6
**Occupation**		
Self-employed	120	27.8
Employed	262	60.7
Unemployed/Housewife	50	11.6
**Gestation week**		
<30 weeks	163	37.7
30–37 weeks	200	46.3
>37 weeks	69	16.0

**Table 2:** Knowledge about SHS.

Characteristics	n	%	95% CI
**Definition of SHS**	268	62.0	57.34; 66.51
**Health effects of SHS on pregnant women**			
Cardiovascular diseases	158	36.6	32.14; 41.24
Pulmonary diseases	398	92.1	89.17; 94.33
Lung cancer	349	80.8	76.78; 84.24
Other cancers	175	40.5	36.0; 45.23
Miscarriage, complications	323	74.8	70.44; 78.66
High blood pressure	110	25.5	21.56; 29.80
Sexual dysfunction	69	16.0	12.80; 19.75
Peptic ulcer	44	10.2	7.66; 13.42
**Health effects of SHS on infants**			
Miscarriage, stillbirth	330	76.4	72.14; 80.17
Preterm birth, low birth weight	332	76.9	72.62; 80.61
Infant mortality	134	31.0	26.81; 35.56
Sudden infant death syndrome	71	16.4	13.22; 20.25
**Facilities which prohibit completely smoking indoor and outdoor according to the law**			
Health care facilities	398	92.1	89.17; 94.33
Education facilities	386	89.4	86.06; 91.93
Childcare facilities	412	95.4	92.92; 97.0
Facilities with high fire risk	398	92.1	89.17; 94.33
**Facilities which prohibit completely smoking indoor according to the law**			
Workplace	328	75.9	71.65; 79.74
Schools	303	70.1	65.63; 74.84
Public areas	249	57.6	52.90; 62.24
Public transportation (bus, fly, train)	377	87.3	83.77; 90.10
	**Mean**	**SD**	
**Knowledge score (0–** **10 points)**	4.19	2.12	3.99; 4.39

**Table 3:** Attitude about SHS.

Characteristic	n	%	95% CI
Smoking at home should not be allowed	370	85.7	82.0; 88.66
Smoking at workplace should not be allowed	304	70.4	65.87; 74.50
Smoking in public areas should not be allowed	292	67.6	63.01; 71.86
	**Mean**	**SD**	
Attitude score (0–10 points)	7.45	3.05	7.10; 7.80

**Table 4:** Practice about SHS.

Characteristic	n	%	95% CI
**Remind smokers at home**			
Always	144	33.3	29.03; 37.93
Usually	116	26.9	22.87; 31.24
Sometimes	81	18.8	15.33; 22.72
Rarely	21	4.9	3.18; 7.35
Never	70	16.2	13.0; 20.0
**Action to stop smoking behavior at home**			
Assertive attitude	131	30.3	26.16; 34.84
Call support of people around	76	17.6	14.27; 21.49
Require smokers to go to a separate room	227	52.6	47.81; 57.23
Do nothing	3	0.7	0.22; 2.14
**Remind smokers at workplace**			
Always	66	15.3	12.17; 19.00
Usually	71	16.4	13.22; 20.25
Sometimes	141	32.6	28.36; 37.22
Rarely	36	8.3	6.07; 11.35
Never	118	27.3	23.30; 31.73
Total	432	100	
**Action to stop smoking behavior at workplace**			
Assertive attitude	57	13.2	10.30; 16.74
Call support of people around	50	11.6	8.87; 14.96
Require smokers to go to a separate room	192	44.4	39.80; 49.18
Report to security/authority	19	4.4	2.82; 6.80
Do nothing	219	50.7	45.97; 55.41
**Remind smokers at public areas**			
Always	11	2.6	1.41; 4.54
Usually	23	5.3	3.56; 7.90
Sometimes	72	16.7	13.42; 20.50
Rarely	63	14.6	11.54; 18.25
Never	263	60.9	56.17; 65.39
**Action to stop smoking behavior in public areas**			
Assertive attitude	19	4.4	2.82; 6.80
Mobilize the support of surrounding people	10	2.3	1.25; 5.25
Require smokers to stay in a separate room	74	17.1	13.85; 21.00
Report to security/authority	6	1.4	0.62; 3.07
Do nothing	341	78.9	74.82; 82.54
	**Mean**	**SD**	
**Practice score (0–10 points)**	4.30	2.39	1.80; 6.81

**Table 5:** Associated factors with knowledge, attitude and practice.

Characteristics	Knowledge Score		Attitude Score		Practice Score	
	Coef. ^1^	95% CI ^2^	Coef.	95% CI	Coef.	95% CI
**Age group**						
18–25 (ref)						
26–30			−0.56 *	−1.13; 0.02		
31–35	0.51 **	0.02; 0.99			−0.47 *	−1.01; 0.08
>35	0.59	−0.13; 1.31				
**Occupation**						
Self-employed (ref)						
Employed	0.66 ***	0.22; 1.11				
Unemployed/Housewife	0.53	−0.18; 1.24			−1.08 ***	−1.76; −0.39
**Living location**						
Urban (ref)						
Rural			0.99 ***	0.24; 1.74	−0.60 **	−1.16; −0.04
**Gestation week**	−0.02 **	−0.03; −0.00	0.02	−0.00; 0.04		
**Source of information**						
News or magazines						
No (ref)						
Yes	0.65 ***	0.22; 1.08				
Advertisement						
No (ref)						
Yes					−1.26 **	−2.39; −0.13
Internet						
No (ref)						
Yes			0.68 **	0.01; 1.36	0.49 *	−0.05; 1.02
Loudspeaker						
No (ref)						
Yes			1.17 **	0.05; 2.28		
Leaflets						
No (ref)						
Yes	0.71	−0.23; 1.66	−0.79	−1.95; 0.37		
**Knowledge score**					0.13 **	0.03; 0.24

*** *p* < 0.01; ** *p* < 0.0; * *p* < 0.1; ^1^ Coefficient; ^2^ Confidence interval.

## Appendix A

**Table A1:** Questionnaire to pregnant women.

No.	Question	Participant’s Choice/Answer
**I. General information**		
A1.	Please inform your age (until the time data is collected)	…… (years old)
A2.	Please choose your highest education attainment	1. Did not go to school 2. Primary 3. Secondary 4. High-school 5. College/university 6. Above university
A3.	Please choose your current occupation	1. Farmer 2. Handicraft 3. Business 4. Lecturer/teacher 5. White-collar 6. Self-employed
A4.	Please choose your current living area	1. Urban 2. Rural
A5.	Please inform your gestation week	1. ……. (weeks) 2. Already born
A6.	Have you ever heard about second-hand smoke (SHS)?	1. Yes 2. No
A7.	Please choose your source of information in case you have heard about SHS (multiple choice)	1. News or magazines 2. Television 3. Radio 4. Advertisement 5. Internet 6. Loudspeaker 7. Posters/banners 8. Leaflets 9. Others (specify):………………………
**II. KAP of SHS**		
B1	What is the definition of SHS?	1. SHS is the contact with the smoke from the cigarette 2. SHS is the contact with the air around the one who smokes 3. SHS is the combination of 1. and 2. 97. No opinion about SHS 98. Other (specify):………………….… 99. Don’t want to answer
B2	Please select all answers that you think are correct for the question “What are the health effects of SHS on pregnant women?” (multiple choice)	1. Cardiovascular diseases 2. Pulmonary diseases 3. Lung cancer 4. Other cancers 5. Low-birth weight infants 6. Miscarriage, complications 7. High blood pressure 8. Sexual dysfunction 9. Peptic ulcer 97. Don’t know 98. Other (specify): ……………………. 99. Don’t want to answer
B3	Please select all answers that you think are correct for the question “What are the health effects of SHS on infants?” (multiple choice)	1. Miscarriage, stillbirth 2. Preterm birth, low birth weight 3. Infant mortality 4. Sudden infant death syndrome 97. Don’t know 98. Other (specify): …………………… 99. Don’t want to answer
B4	Please select all answers that you think are correct for the question “What are the facilities which prohibit completely smoking indoors and outdoors according to the law?” (multiple choice)	1. Health care facilities 2. Education facilities 3. Childcare facilities 4. Facilities with high fire risk 97. Don’t know 99. Don’t want to answer
B5	Please select all answers that you think are correct for the question “What are the facilities which prohibit completely smoking indoors according to the law?” Law No. 09/2012/QH13) (multiple choice)	1. Workplace 2. Schools 3. Public areas 4. Public transportation (bus, fly, train) 97. Don’t know 99. Don’t want to answer
B6	Do you think that smoking at home should be allowed?	1. Allowed 2. Not allowed but acceptable 3. Not allowed 4. There is no regulation about this in the law 97. Don’t know 99. Don’t want to answer
B7	Do you think that smoking at workplace should be allowed?	1. Allowed 2. Not allowed but acceptable 3. Not allowed 4. There is no regulation about this in the law 97. Don’t know 99. Don’t want to answer
B8	Do you think that smoking in public areas should be allowed?	1. Allowed 2. Not allowed but acceptable 3. Not allowed 4. There is no regulation about this in the law 97. Don’t know 99. Don’t want to answer
B9	How frequently do you remind smokers at home?	1. Always 2. Usually 3. Sometimes 4. Rarely 5. Never
B10	What do you do to stop smoking behavior at home?	1. Assertive attitude 2. Call support of people around 3. Require smokers going to separated room 4. Do nothing 98. Other (specify):……………………..
B11	How frequently do you remind smokers at workplace?	1. Always 2. Usually 3. Sometimes 4. Rarely 5. Never
B12	What do you do to stop smoking behavior at workplace?	1. Assertive attitude 2. Call support of people around 3. Require smokers going to separated room 4. Report to security/authority 5. Do nothing 98. Other (specify):………………………
B13	How frequently do you remind smokers at public areas?	1. Always 2. Usually 3. Sometimes 4. Rarely 5. Never
B14	What do you do to stop smoking behavior at public areas?	1. Assertive attitude 2. Call support of people around 3. Require smokers going to separated room 4. Report to security/authority 5. Do nothing 98. Other (specify):……………………..
